# CpoS-Inc interactions facilitate host cell modulation during *Chlamydia trachomatis* infection

**DOI:** 10.1128/iai.00548-25

**Published:** 2025-11-18

**Authors:** Xavier Tijerina, C.A. Jabeena, Robert Faris, Zhen Xu, Parker Smith, Nicholas J. Schnicker, Mary M. Weber

**Affiliations:** 1 Department of Microbiology and Immunology, University of Iowa Carver College of Medicine 12243 , Iowa City, Iowa, USA; 2 Protein and Crystallography Facility, University of Iowa Carver College of Medicine 12243 , Iowa City, Iowa, USA; University of California, Davis, California, USA

**Keywords:** Inc-Inc interactions, T3S, *Chlamydia trachomatis*, CpoS, InaC

## Abstract

** ***Chlamydia trachomatis* (*C.t*.), the leading bacterial cause of sexually transmitted infections, replicates within a unique intracellular compartment called the inclusion, which is modified by secreted proteins known as inclusion membrane (Inc) proteins. Here, we further characterize CpoS, an Inc protein previously shown to be critical for bacterial replication and inclusion development. We demonstrate that CpoS directly binds multiple coiled-coil region-containing Incs and engages Rab GTPases at a separate site. Notably, CpoS-InaC interactions facilitate the recruitment of select Arf GTPases to the inclusion membrane, while Rab recruitment occurs independently of these interactions. Biochemical and biophysical analyses revealed that Incs self-oligomerize to form higher-ordered structures, with CpoS adopting a tetrameric conformation resembling that of eukaryotic SNARE proteins. We propose that these assemblies serve as scaffolds to orchestrate vesicle docking, tethering, and fusion. Our findings highlight the intricate interplay between bacterial and host factors, revealing how *C.t*. leverages both Inc-Inc interactions and host protein engagement to manipulate vesicular trafficking and sustain infection.

## INTRODUCTION

*Chlamydia trachomatis* (*C.t*.) is the most common bacterial cause of sexually transmitted infection and the leading cause of infectious blindness worldwide. *C.t*. is divided into multiple serovars, separated by variations in the Major Outer Membrane Protein (MOMP), with subsets associated with either ocular or genital infections (1, 2). Genital infections caused by *C.t*. are often asymptomatic, leading to prolonged infections with severe complications, such as pelvic inflammatory disease, infertility, ectopic pregnancy, and an increased risk of cervical and ovarian cancer (3–6). Although these infections can be treated with antibiotics, reinfection is common due to a lack of long-term immunity and the absence of a vaccine (7–9). A deeper understanding of the cellular and molecular mechanisms that *C.t*. employs to cause disease is crucial for developing improved therapeutics and guiding vaccine development.

Chlamydial replication requires the formation of a privileged niche, termed the inclusion, which is extensively modified early in infection through the incorporation of unique type III secreted effector proteins known as inclusion membrane proteins (Incs) (10, 11) into the membrane-bound vacuole. Thirty-seven Inc proteins have been confirmed to localize to the inclusion membrane during infection, representing 5% of *C.t*.’s highly reduced genome (12). Given their strategic positioning at the host-pathogen interface, Incs are integral to mediating fusion with host-derived vesicles, forming membrane contact sites with host organelles, redirecting host vesicular transport to the inclusion, and modulating the host cell cytoskeleton (13–22), all of which demands not only controlled interactions with host proteins but is also hypothesized to require interactions with other Inc proteins (23, 24). The retention of a large number of Inc proteins suggests that they play a critical role in the intracellular survival of *Chlamydia*. Indeed, recent work from our laboratory (25) and others (21, 22) revealed that the absence of select Inc proteins, including CTL0481 (CT229 in serovar D), results in premature inclusion lysis and host cell death, leading to its designation as a Chlamydia promoter of Survival (CpoS). CpoS binds to and recruits multiple host Rab GTPases to regulate host trafficking pathways and suppresses cell-autonomous immunity (14, 16, 17, 21, 22, 26). CpoS also interacts with other Inc proteins, including IPAM, CT222, and IncD (21–23). However, the functional significance of these Inc-Inc interactions remains poorly understood.

To acquire key nutrients from the host and membrane for the growing inclusion, *C.t*. modulates vesicular trafficking and fusogenicity with the inclusion membrane (11, 27). Small guanosine triphosphate (GTP)-binding proteins, such as Rab GTPases and Arf GTPases, regulate key aspects of vesicle trafficking, including vesicle formation, transport, tethering, and fusion (28–30) . Rab GTPases interact directly or indirectly with soluble NSF (N-ethylmalemide-sensitive factor) attachment receptors (SNAREs) (31) to regulate the assembly or disassembly of SNARE complexes (32, 33). In addition to hijacking small GTPases to modulate host vesicular transport, *C.t*. Inc proteins engage host SNARE proteins (34–36). Furthermore, bioinformatic analysis of Inc proteins has revealed that at least three of them (IncA, IPAM, and InaC) possess SNARE-like domains (SLD) (37) that mediate homotypic inclusion fusion (18, 36, 38, 39). In eukaryotes, SNARE domains form heteromultimers with other SNARE domains. Typically, this involves one R-SNARE associating with three Q-SNAREs across the donor and target membrane, in addition to Rab GTPases and tethering factors (33, 40, 41). Upon vesicle docking, the SNARE domain aligns to form a stable four-helix bundle, driving the zippering of donor and target membrane leaflets resulting in membrane fusion. A crucial feature of the SNARE domain is its highly conserved central ionic “0” layer, containing either a conserved arginine (R-SNARE) or glutamine (Q-SNARE). The salt bridges formed by the ionic layer, combined with hydrophobic interactions from surrounding leucine (heptad) repeats, initiate the zippering process that drives membrane fusion.

While the engagement of SNARE proteins and small GTPases is critical for modulating vesicular trafficking during *C.t*. infection, interactions among inclusion membrane proteins may play an equally vital role in promoting vesicle tethering and fusion (42). Advances in chlamydial genetics, along with new tools to capture dynamic protein-protein interactions (PPIs), suggest that Inc proteins can bind to one another throughout the developmental cycle (21, 22, 24). Notably, CpoS forms higher oligomeric structures, such as tetramers, which we posit enables it to bind to both other Incs and host factors. Collectively, our work reveals that Inc-Inc interactions add to the complex organization of the inclusion membrane and collaboration between these bacterial proteins may enable the pathogen to more efficiently subvert host regulations. Significantly, we show that disruption of these interactions can perturb host-pathogen interactions, underscoring the importance of these bacterial effector interactions.

## MATERIALS AND METHODS

### Bacteria and cell culture

*C.t.* serovar L2 (LGV 434/Bu) was propagated in HeLa 229 cells (American Type Culture Collection) grown in RPMI 1640 with L-glutamine (ThermoFisher Cat#11875-093) supplemented with 10% Fetal Bovine Serum (FBS) (VWR Cat#89510-186), 1 mM sodium pyruvate (ThermoFisher Cat#11360070), and sodium bicarbonate. Cells were grown at 37°C in a humidified incubator with 5% CO_2_. When required, EBs were purified from HeLa cells using a gastrografin density gradient (43).

### Cloning

For expression in *C.t*., CpoS and various coiled-coil (CC)-containing Incs were PCR amplified from purified *C.t*. L2 genomic DNA, and a C-terminal HA-tag or FLAG-tag was added. The resulting PCR fragments were digested with KpnI-HF (NEB Cat# R3142L) and SalI-HF (NEB Cat# R3138L) and cloned into pBomb4. To truncate or specifically delete the CC regions, the regions determined using SMART sequence analysis were removed using C-terminal truncations or internal deletions conducted by GenScript. For *in vitro* biochemical characterization, full-length (FL) or Inc proteins lacking the transmembrane domain were cloned into the pMALc-5VT vector (Protein and Crystallography Facility, UIOWA) using NotI (NEB Cat#R3189L) and SalI-HF, resulting in an N-terminal fusion to an MBP-tag and a C-terminal 6×-His-tag. Truncated CpoS was cloned into pGEX6P1 (Sigma Cat#GE28-9546-48) using BamHI-HF (NEB Cat#R3136L) and XhoI (NEB Cat#R0146L) to generate an N-terminal fusion to a GST-tag. All primers are listed in Table S1. The integrity of all constructs was confirmed by sequencing (McLab).

### Immunoprecipitation

HeLa cells were infected at a multiplicity of infection (MOI) of 3 with *C.t*. L2 pBomb4-tet-CpoS HA-tag and one of the various pBomb4-tet-Inc FLAG-tag strains. Alternatively, HeLa cells were transfected with pcDNA3.1eGFP-Rab14 (Genscript Cat#OHu12700) or pcDNA3.1 eGFP Rab35 (Genscript Cat#OHu12018) followed by infection at an MOI of 3 with *C.t*. L2 pBomb4-tet-CpoS HA-tag and one of the various pBomb4-tet-Inc FLAG-tag strains. Expression of both *C.t*. proteins was induced with 10 ng/mL anhydrous tetracycline (aTc) at the time of infection. At 24 hpi, cells were lysed on ice in eukaryotic lysis solution (ELS) (50 mM Tris HCl, pH 7.4, 150 mM NaCl, 1 mM EDTA, and 1% Triton-X 100) and spun at 12,000 *× g* for 20 min. Supernatants were incubated with HA beads (anti-HA Magnetic beads, Thermo Fisher Scientific Cat#88836) for 1-2 h at 4 ˚C. For CpoS truncations, FLAG beads were used for IP and incubated for 2 hours at 4 ˚C. The ligand-bound beads were washed 5 times with ELS without Triton-X 100. Proteins were eluted from the beads in NuPAGE LDS Sample Buffer (Thermo Fisher Scientific Cat#NP0007) by heating at 100°C for 5 min prior to analysis by western blotting.

### Western blotting

Samples were separated by sodium dodecyl sulfate-polyacrylamide gel electrophoresis (SDS-PAGE) on a 4-12% Bis-Tris protein gel (GenScript Cat#M00653) with MES running buffer. Proteins were transferred onto PVDF membranes and blocked in 5% milk in Tris-buffered saline with Tween 20 overnight at 4 ˚C. Membranes were probed with anti-FLAG (Thermo Fisher Scientific Cat#701629) or anti-HA (1:1500 Millipore Sigma Cat#SAB2702217) primary antibodies and goat anti-rabbit HRP conjugate (1:10,000 BioRad Cat#1706515) secondary antibody.

### Super-resolution microscopy

HeLa cells, seeded on #1.5 High Precision Glass Coverslips (Bioscience Tools Cat#CSHP-No1.5-13), were infected at an MOI of 5 with *C.t*. L2 pBomb4-tet-CpoS HA-tag and one of the various pBomb4-tet-Inc FLAG-tag strains. Expression of both proteins was induced with 10 ng/mL aTc at the time of infection. At 24 hpi, cells were fixed in 2% formaldehyde and permeabilized with 0.1% Triton-X 100. Samples were blocked with 3% BSA in phosphate-buffered saline (PBS) followed by a 1-h incubation with primary antibodies, rabbit anti-HA (1:1000, Novus Cat#NB600-363), and mouse anti-FLAG (1:1000, Sigma Cat#F2555), prepared in blocking buffer. Cells were washed with PBS followed by incubation with the secondary antibodies goat anti-mouse DyLight594 (1:250, Invitrogen Cat#35561) and goat anti-rabbit STAR635 (1:250, Abberior Cat#ST635P-1002) prepared in blocking buffer. Coverslips were washed with PBS and mounted on glass slides using ProLong Diamond Antifade Mountant (Invitrogen Cat#36961) and allowed to cure for 24 h before imaging. STED images were taken using a Leica SP8 inverted microscope. Images were deconvoluted using Imaris Professional Software.

### Confocal microscopy

HeLa cells, seeded on coverslips, were transfected with GFP-tagged Rab (mentioned above) and Arf GTPases (Addgene #39554, #39556) using Lipofectamine LTX. At 24 h post-transfection, cells were infected with *C.t*. L2 WT, *cpoS::bla* (14, 25), *CT228:bla,* or *InaC::bla* at an MOI of 2. At 24 hpi, cells were fixed with methanol and blocked with 3% BSA in PBS. The inclusion membrane was stained with either anti-IncB or anti-IncA antibodies (1:250) (kindly provided by Ted Hackstadt, PhD) diluted in blocking buffer. Cells were washed with PBS and incubated with goat anti-rabbit AlexaFluor594 secondary antibody (1:1,000, Invitrogen) and DAPI (1:1,000, Invitrogen Cat#D1306) in blocking buffer. After washing with PBS, cells were mounted on glass slides using ProLong Diamond Antifade Mountant and allowed to cure for 24 h before imaging. Confocal images were taken using a Nikon inverted microscope. Pearson correlation coefficients (R value) were calculated using ImageJ Coloc2 function of entire intact inclusions.

### Protein expression

Sequence-confirmed plasmids were transformed into BL21 (DE3) or Rosetta (DE3) cells, grown at 37°C to an OD600 of 0.6, induced with 1 mM isopropyl β-D-1-thiogalactopyranoside (IPTG) (Research Products International Cat#I56000-25.0), and incubated overnight at 18°C with shaking to promote proper folding of the soluble proteins. For purification of MBP-tagged proteins, cells were pelleted and resuspended in sonication buffer (20 mM Tris-HCl, pH 7.5, 200 mM NaCl, 1 mM DTT, 1 mM EDTA, and 10% glycerol) containing a protease inhibitor cocktail (Millipore Sigma Cat#1187350001) and DNase I (Millipore Sigma Cat#10104159001). Cells were lysed by sonication (Sonics Vibram-Cell) at 38% amplitude with 1 s on/1 s off pulses. The soluble fraction was collected by high-speed centrifugation at 12,000 *× g* for 1 h and applied to an Amylose resin high-flow column (NEB Cat#E8022S). Unbound proteins were removed by washing with sonication buffer, and bound proteins were eluted with sonication buffer containing 10 mM maltose (Research Products International Cat#M22000).

For purification of GST-CpoS C-ter, cells were lysed in lysis buffer (50 mM Tris-HCl, pH 7.5, 500 mM NaCl, 10% glycerol, 1 mM DTT, 1X PIC, and 5 µL of 50 units of DNase I). The supernatant was applied to glutathione agarose beads (Thermo Fisher Scientific Cat#16100), washed with wash buffer (50 mM Tris-HCl, pH 7.5, 300 mM NaCl, 10% glycerol, 1 mM DTT), and eluted with elution buffer (50 mM Tris-HCl, pH 7.5, 150 mM NaCl, 10% glycerol, 10 mM reduced glutathione). Protein samples were resolved using 4%–12% Bis-Tris SDS-PAGE gel and stained with Coomassie Brilliant Blue (Bio-Rad Cat#1610436). Protein identity was confirmed by Western blotting using anti-GST (1:5,000, Thermo Fisher Scientific Cat#MA4-004) or anti-MBP (1:5,000, Santa Cruz Biotechnology Cat#sc-13564) antibodies. Proteins were further purified using a 24 mL Analytical Superdex 200 Increase 10/300 Gl gel-filtration column (GE Healthcare) with 20 mM Tris, pH 7.5, 200 mM NaCl, 10% glycerol, and 1 mM DTT. Injection volume was 500 µL with a flow rate of 0.5 ml/min, and fractions were collected in 500 µL volumes. Purified protein was concentrated using Amicon ultra-centrifugal filter devices (Millipore Sigma Cat#UFC801024, 10 kDa cutoff) and assessed again using SDS-PAGE gel.

### *In vitro* binding assays using GST-pulldown

To assess *in vitro* binding, 500 µL of glutathione agarose resin was equilibrated with 10 mL of column buffer (50 mM Tris, pH 7.4, 500 mM NaCl, 10% glycerol, 1 mM DTT). Twenty micrograms of GST-tagged CpoS C-ter were added and incubated for 2 h at 4°C. After three washes with column buffer, 20 µg of MBP-tagged Incs (MBP-InaC C-ter, MBP-IPAM C-ter, MBP-CT449, or MBP control) was added and incubated overnight at 4°C with rotation. The column was washed three times with wash buffer to remove unbound proteins. The protein complex was eluted by incubating the resin with a GST elution buffer for 30 min. The flow-through was collected, and the complexes were confirmed by Western blotting with anti-GST and anti-MBP antibodies and goat anti-mouse HRP conjugated secondary antibody. Results were obtained from at least three independent experiments.

### Mass photometry

To determine the oligomeric states of CpoS in solution and accurately measure molecular mass, mass photometry (MP) was performed using a Refyn TwoMP mass photometer (Refeyn Ltd, Oxford, UK). Microscope coverslips (24 × 50 mm, Thorlabs Inc. Cat#CG15KH) and silicon gaskets (Grace Bio-Labs Cat#103250) were cleaned by serial rinsing with Milli-Q water and HPLC-grade isopropanol (Sigma Aldrich Cat#34863) followed by drying with a filtered air stream. All MP measurements were performed at room temperature using Dulbecco’s phosphate-buffered saline (DPBS) without calcium and magnesium (Gibco Cat#14200-075). The instrument was calibrated using a protein standard mixture: β-amylase (Sigma-Aldrich Cat#A7005-10KU, 56, 112, and 224 kDa) and thyroglobulin (Sigma-Aldrich Cat#T1001-100MG, 670 kDa). In brief, 15 µL of DPBS buffer was placed in the well to find focus before each measurement. The focus position was locked using the default droplet-dilution autofocus function, after which 5 µL of protein (100 nM) was added and briefly mixed before movie acquisition started. Movies were acquired for 60 s (3,000 frames) using AcquireMP (Refeyn Ltd) under standard settings. All movies were processed and analyzed using DiscoverMP (Refeyn Ltd.) and the interferometric contrast values were converted to molecular masses using the calibration function with protein standards. MP data were plotted as histograms or kernel density estimate (KDE) distributions. The distribution peaks were fitted with Gaussian functions to obtain the average molecular mass of each distribution component.

### Size exclusion chromatography coupled with multi-angle light scattering and small-angle X-ray scattering (SEC-MALS-SAXS)

For detailed biophysical analysis of CpoS proteins in solution, SEC-MALS-SAXS was performed. Datasets were collected using the 18-ID-D BioCAT Beamline at the Advanced Photon Source (APS) at Argonne National Laboratory (Chicago, IL). Samples were centrifuged for 5 min at 13,000 rpm to remove any potential aggregates prior to column loading. Samples containing 1 mg/mL of MBP-tagged CpoS in 250 µL were injected onto a 24 mL Superose 6 Increase 10/300 Gl column (GE) equilibrated with buffer (20 mM Tris, pH 7.5, 50 mM NaCl, 1 mM EDTA, 2% glycerol, and 1 mM DTT) at a flow rate of 0.6 mL/min on an Agilent 1300 chromatography system. Column eluant was analyzed in line by the UV absorbance detector of the Agilent 1300 chromatography system, then directed into the DAWN Heleos-II light scattering (LS) and OptiLab T-rEX refractive index detectors in series. Finally, the elution trajectory directed samples into a 1.0-mm ID quartz capillary SAXS sample cell. Scattering data were collected every 1 s using a 0.3-s exposure and detected with an Eiger2 XE 9M pixel detector (DECTRIS) with a 12 keV (1.033 Å wavelength) X-ray beam covering a q-range of 0.0027 < q < 0.41 Å^−1^ (q = 4π*sinθ/λ, where λ is the X-ray wavelength, and 2θ is the scattering angle). Accurate protein molecular weights from MALS data were determined using the ASTRA software (Wyatt Technology).

### SAXS data processing and modeling

SAXS data reduction, buffer subtraction, and further analysis were performed using BioXTAS RAW version 2.1.4 (44). An average of 30 frames before and after the eluted peaks was used for buffer subtraction. Protein peaks were also run through evolving factor analysis (EFA) to deconvolute peaks into the individual scattering components where applicable (45). The forward scattering intensity I(0) and radius of gyration (Rg) were calculated from the Guinier fit. The normalized Kratky plot was generated in the BioXTAS RAW software (46), and pair distance distribution *P*(r) plot was calculated using the program GNOM embedded in the BioXTAS RAW software (46).

### Statistical analyses and densitometry

When necessary, statistical analysis was performed using GraphPad Prism 9.3.0 software. One-way ANOVAs were used followed by Tukey’s multiple comparisons with *P* < 0.05 (*), *P* < 0.01 (**), and *P* < 0.001 (***), and *P* < 0.0001 (****).

## RESULTS

### CpoS binds to multiple Inc proteins

**Fig 1:**
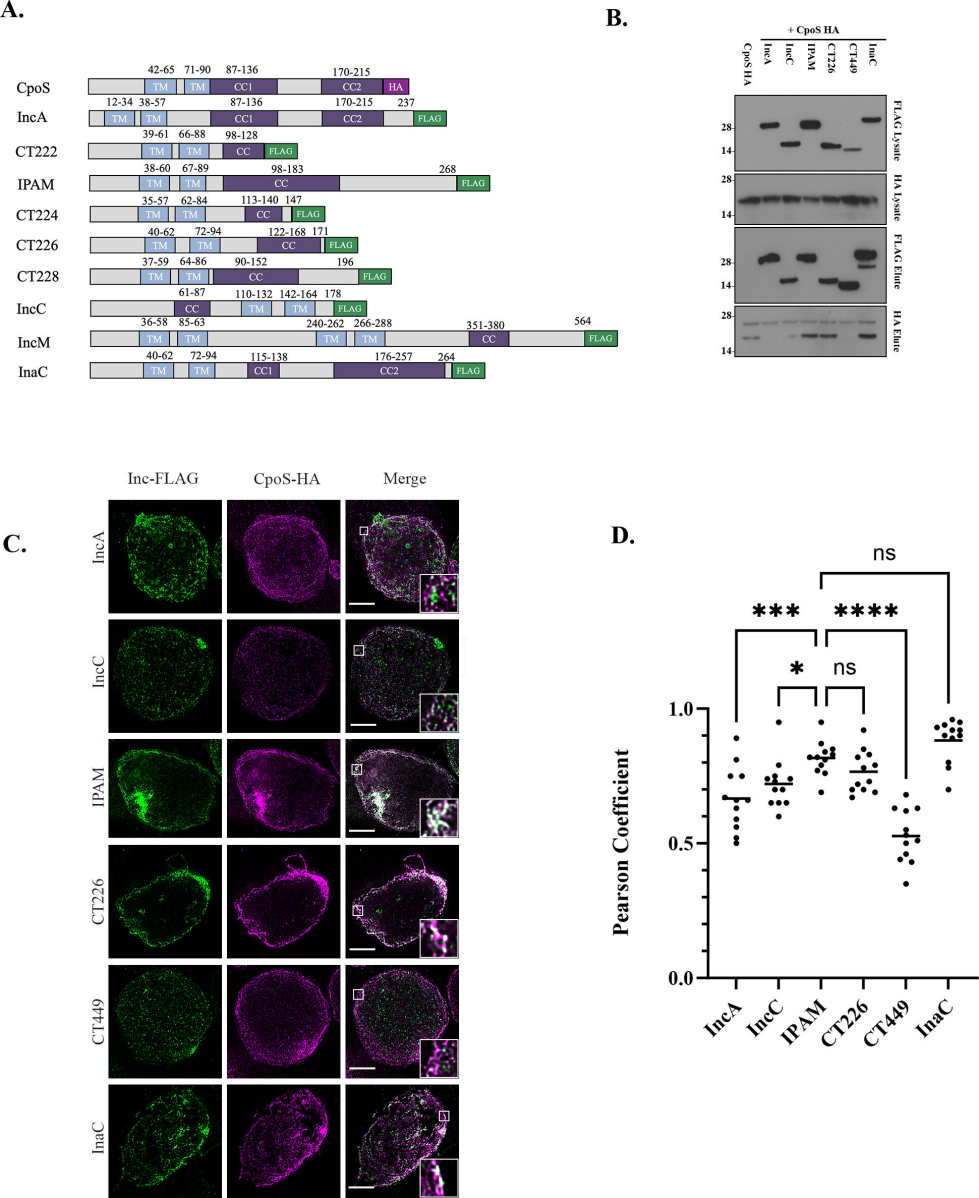
CpoS binds to multiple coiled-coil region containing Incs. (**A**) Schematic of Inc proteins. Bi-lobed hydrophobic domain is shown in blue and the coiled-coil region is represented in purple. HA tag is shown in magenta and FLAG tag in green. (**B and C**) HeLa cells were coinfected at an MOI of 2.5 with HA-tagged CpoS and FLAG-tagged Inc for 24 h. (**B**) FLAG-tagged Incs were immunoprecipitated from cell lysates using FLAG-magnetic beads, and samples were analyzed by Western blotting. MW- IncA FLAG-tag 28.5 kDa, IncC FLAG-tag 19.6 kDa, IPAM FLAG-tag 30.6 kDa, CT226 FLAG-tag 19.3 kDa, CT449 FLAG-tag 12 .kDa, InaC FLAG-tag 30.5 kDa, and CpoS HA-tag 24.6 kDa. (**C**) Cells were formaldehyde fixed 24 hpi and processed for super-resolution microscopy using anti-HA and anti-FLAG antibodies with anti-mouse 594 and anti-rabbit 635 secondaries, respectively. Scale bar denotes 5 µm. White boxes denote the area used for inset. (**D**) Pearson correlation coefficients (R value) were calculated using ImageJ Coloc2 function. The graph is representative of 10 images per experiment. Dashed line represents cutoff for significant colocalization (0.70). (**B and C**) Data are representative of three independent experiments.

CpoS was previously confirmed to bind to IPAM during infection (21). However, we hypothesized that it may engage other Inc proteins throughout the chlamydial lifecycle. As coiled-coil (CC) domains are found in numerous proteins and mediate important PPIs that control diverse cellular processes, we sought to determine whether CpoS might bind to CC-containing Incs. Using the Simple Modular Architecture Research Tool (SMART), we evaluated the 37 Inc proteins previously shown to localize to the inclusion membrane (47). In line with previous reports (37), we identified nine Inc proteins along with CpoS that possess a CC region: IncA, CTL0476/CT222, CTL0476/CT223 (IPAM), CTL0477/CT224 (Tri1), CTL0478/CT226, CTL0480/CT228, CTL0485/CT233 (IncC), CTL0540/CT288 (IncM), and InaC (Fig. 1A).

Due to a lack of commercially available Inc antibodies, we used a co-infection model (48) in which HeLa cells were infected with two strains: *C.t*. CpoS-HA and another Inc fused to a FLAG-tag. Since inclusions fuse, this model results in a single inclusion per cell with both Incs present on the same inclusion membrane (21). Using anti-FLAG magnetic beads, we enriched for the Inc proteins and probed immunoblots with anti-FLAG to confirm the IP and anti-HA to detect interactions with CpoS. Here we demonstrate that CpoS binds to IPAM, CT226, and InaC (Fig. 1B). Due to low expression of CT222, Tri1, CT228, and IncM, we could not accurately ascertain whether they similarly bound to CpoS, and thus they were excluded from subsequent analysis. CT449, an Inc that does not contain a predicted CC region, was used throughout as a negative control to show CpoS interacts with specific Incs.

We next sought to confirm that CpoS co-localizes with these Inc proteins. Using STED super-resolution microscopy to acquire high-resolution images of the inclusion membrane, we demonstrate that CpoS co-localizes with the CC-containing Incs IPAM, CT226, and InaC (Fig. 1C). Because Incs are restricted to the inclusion membrane, some degree of colocalization is expected. To establish a positive control, we used the well-documented interaction between CpoS and IPAM. This colocalization score served as our threshold for significant interaction, with a substantial reduction interpreted as a lack of colocalization with CpoS. Consistent with this, we observed a significant decrease in CpoS colocalization with IncA, IncC, and CT449 (Fig. 1D), none of which co-immunoprecipitated with CpoS (Fig. 1B). Taken together, these results support the hypothesis that CpoS interacts with multiple CC-region-containing Incs during *C.t*. infection.

### CpoS interacts with the C-terminal region of Incs

**Fig 2:**
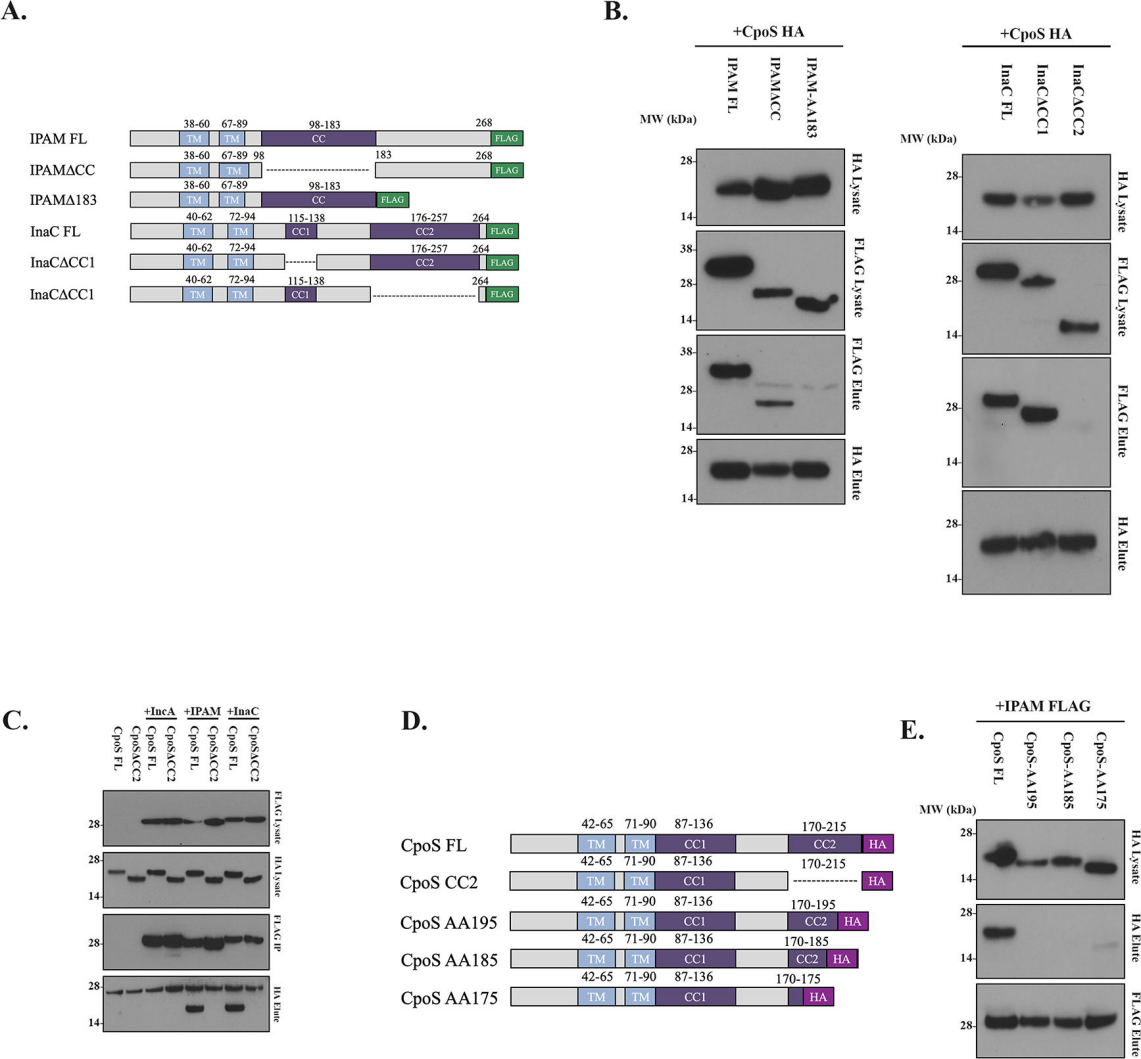
The C-terminus of Incs is required for CpoS-Inc interaction. (**A**) Schematic of Inc truncations. (**B**) HeLa cells were coinfected at an MOI of 2.5 with HA-tagged CpoS and FLAG-tagged Inc for 24 h. CpoS was immunoprecipitated from cell lysates using HA-magnetic beads, and samples were analyzed by Western blotting. MW- IPAM FL FLAG-tag 30.6 kDa, IPAMΔCC FLAG-tag 21.6 kDa, IPAMΔ183-268 FLAG-tag 20.7 kDa, InaC FL FLAG-tag 30.5 kDa, InaC FL FLAG-tag 27.4 kDa, InaCΔCC1 FLAG-tag 30.5 kDa, InaCΔCC2 FLAG-tag 21.0 kDa, and CpoS HA-tag 24.6 kDa. (**C**) HeLa cells were coinfected at an MOI of 2.5 with either CpoS FL-HA or CpoS-ΔCC2-HA and FLAG-tagged Inc for 24 h. The tagged Incs were immunoprecipitated from cell lysates using FLAG-magnetic beads, and samples were analyzed by Western blotting. (**D**) Schematic of sequential truncations of CC2 in CpoS. (**E**) HeLa cells were coinfected at an MOI of 2.5 with the HA-tagged CC2 truncations and FLAG-tagged IPAM for 24 h. IPAM was immunoprecipitated from cell lysates using FLAG-magnetic beads, and samples were analyzed by Western blotting. MW- CpoS HA-tag 24.6 kDa, CpoS HA AA195-tag 22.8 kDa, CpoS HA AA185-tag 21.2 kDa, and CpoS HA AA175-tag 20.0 kDa. (**B, C, and E**) Data are representative of three independent experiments.

Given the crucial role of CC domains in mediating PPIs, we hypothesized that this region is necessary for binding to CpoS. To test this, we created truncation constructs of two Inc proteins, IPAM and InaC, resulting in versions either lacking only the C-terminus or the CC region (Fig. 2A). For InaC, which contains two CC regions, two constructs were generated: one lacked only CC1, while the other lacked CC2 (Fig. 2A). We confirmed these large truncation constructs still localized to the inclusion membrane (Fig. S1). Cells were co-infected with a *C.t*. strain expressing either the FL CpoS-HA or the truncated with the CC-deletion FLAG-tagged Incs. As shown in Fig. 2B, deletion of the C-terminus, but not the CC region from IPAM, resulted in the loss of CpoS binding. In contrast, deletion of CC2 led to a loss of binding to CpoS for InaC, while CC1 was dispensable for the interaction. Collectively, our results indicate that CpoS binding to CC region-containing Incs is context-dependent, requiring specific structural features, with different regions contributing variably to the interaction.

### The CC2 region of CpoS is crucial for mediating Inc-Inc interaction

Previous studies demonstrated that the CC2 region of CpoS interacts with IPAM (21). Building on these findings, we investigated the necessity of the CC2 region interactions with other Inc proteins. We generated CpoS constructs lacking either CC1 and CC2 and confirmed that while CC1 is essential for Rab binding, CC2 appears to be dispensable (Fig. S2). We then conducted co-infection immunoprecipitation assays using either FL CpoS or a CpoS construct lacking CC2, co-infected with FLAG-tagged IPAM, IncA, or InaC. Anti-FLAG beads were used to enrich the CC-Incs and assess their interaction with the different CpoS constructs. As anticipated, we observed a significant decrease in the retention of CpoS-ΔCC2 compared with FL CpoS for each Inc IP (Fig. 2C), indicating that the CC2 region plays a vital role in facilitating interactions with multiple Inc proteins.

Next, we sought to pinpoint the specific region within CC2 required for these interactions. Sequential 10-amino-acid truncations of the CC2 region were generated (Fig. 2D), and co-infection IP assays were repeated to identify truncations that disrupted binding. Our results indicate that the last 20 amino acids of CC2 are required for binding (Fig. 2E). Taken together, these results demonstrate that the c-terminus of CpoS, IPAM, and InaC are involved in Inc-Inc interactions, while the internal regions may be dedicated to interactions with host proteins.

### CpoS binds to both rab GTPases and other Inc proteins

**Fig 3:**
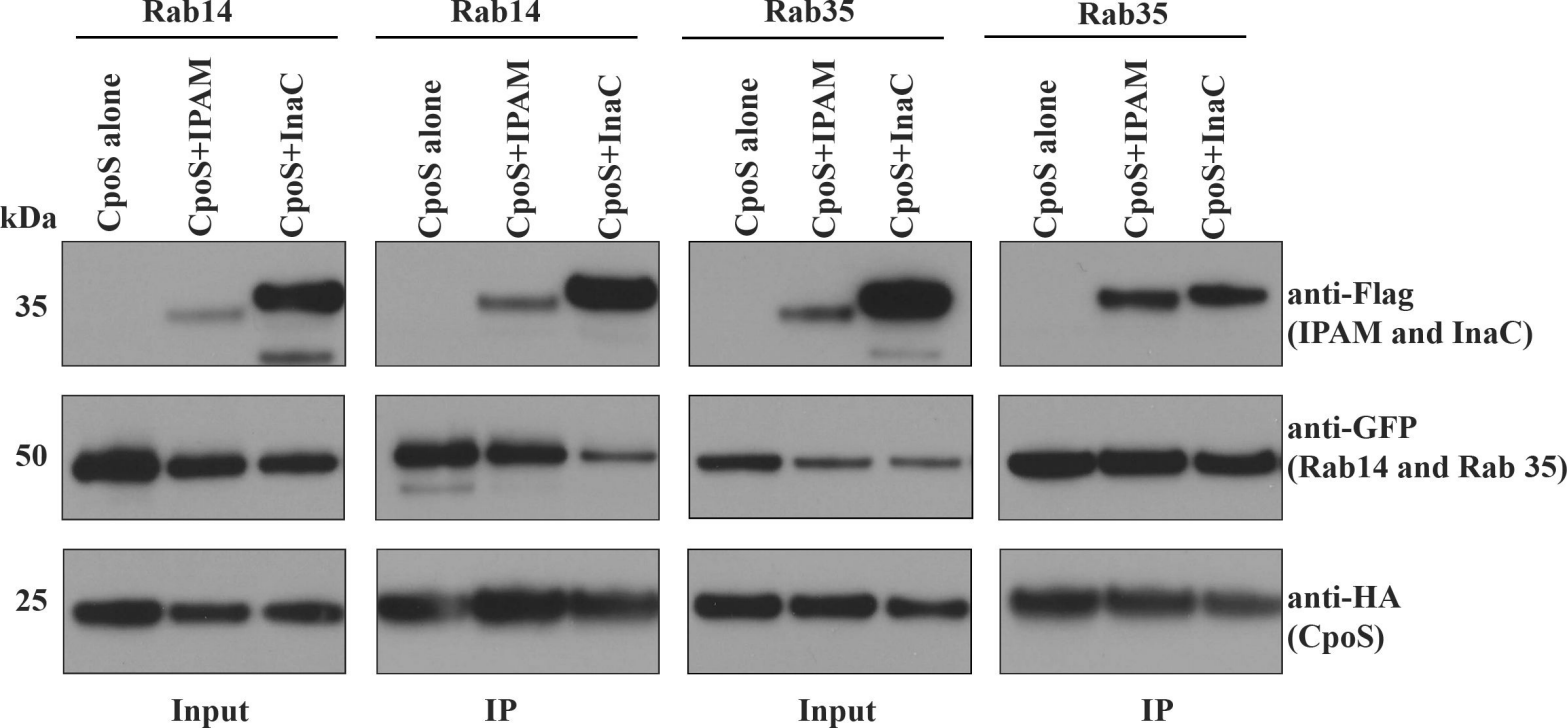
CpoS interacts with Rab GTPases and other *C.t*. Incs. HeLa cells were transfected with pcDNA 3.1 eGFP-Rab14 or pcMV eGFP Rab 35 and co-infected at an MOI of 5 with *C.t.* expressing CpoS-HA and either *C.t.* expressing IPAM-Flag or InaC-Flag. After 24 hpi, CpoS was immunoprecipitated using HA beads, and the elutions were resolved on SDS-PAGE, followed by Western blotting with HA, GFP, and Flag antibodies. Data are representative of three independent experiments.

CpoS contains two coiled-coil regions, with CC1 implicated in binding to Rab GTPases and CC2 mediating interactions with multiple Inc proteins (Fig. 2) (21). This dual-region structure suggests that CpoS may serve as a molecular hub coordinating interactions between host Rab GTPases and other bacterial Inc proteins. To test this hypothesis, we transfected HeLa cells with GFP-tagged Rab14 and Rab35 and used our Inc co-infection model to evaluate Inc and Rab binding to CpoS. Immunoprecipitation of CpoS-HA revealed that it associates with Rab GTPases and other Inc proteins as both populations were detected (Fig. 3). These findings suggest that CpoS plays a central role in linking multiple host and bacterial factors, potentially facilitating the dynamic regulation of inclusion membrane function during infection.

### Tetramer formation by CpoS requires CC2

**Fig 4:**
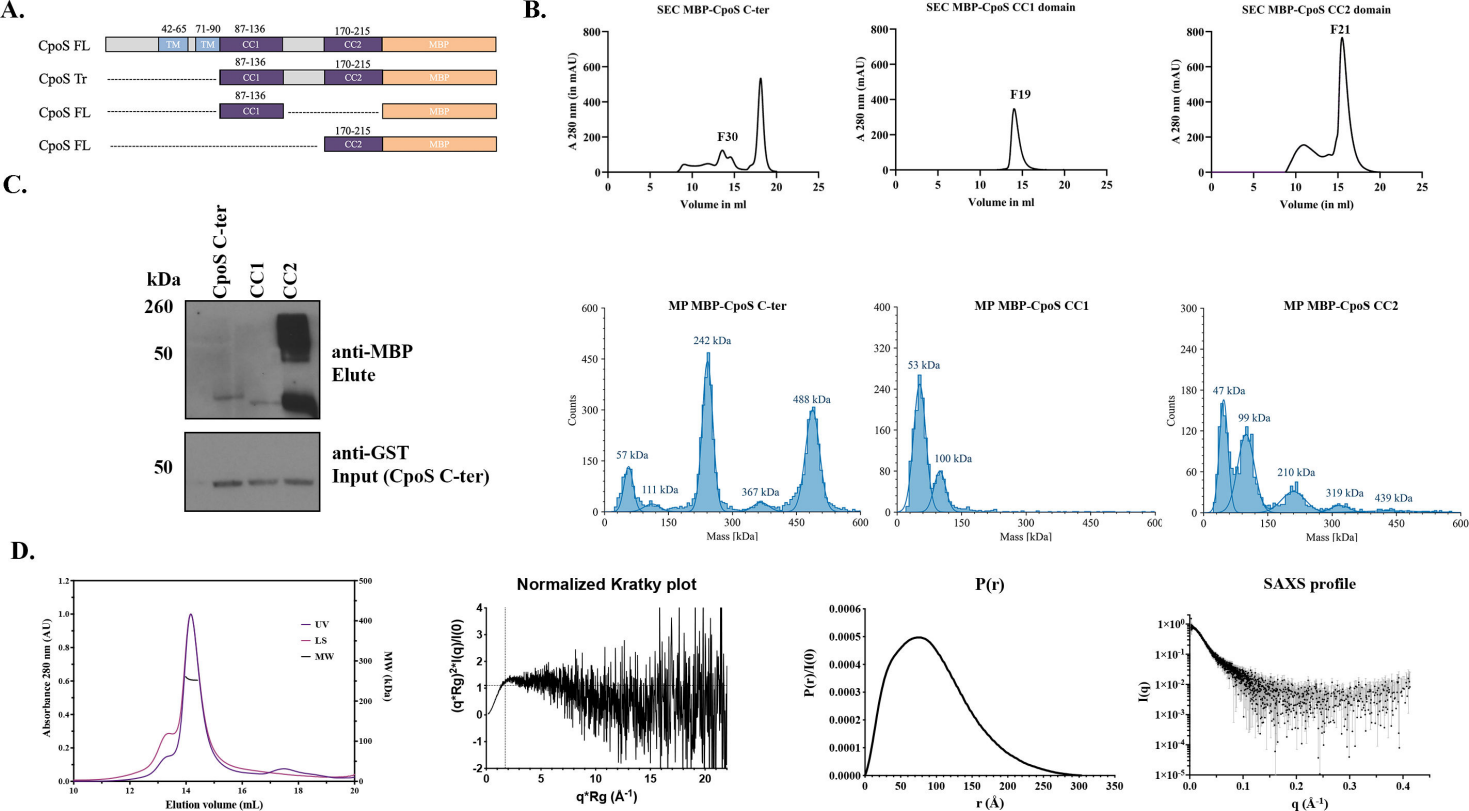
Oligomerization and tetramer formation of the CpoS C-ter region. (**A**) Region organization of various truncations of CpoS protein used in the study. MBP-tagged fusion proteins of CpoS C-ter, CC1 region, and CC2 region were expressed in BL21 (DE3) cells for all the *in vitro* biophysical studies. (**B**) SEC chromatogram of 500 µL (injection volume) of CpoS C-ter on AS 200 column with a flow rate of 0.5 mL/min. Mass photometry histograms of CpoS C-ter (eluted from SEC column, fractions corresponding to 13 and 14 mL) confirmed the dominating tetramers and octamers of the CpoS C-ter proteins in solution and oligomerization of CC2 region (Fractions corresponding to 16 mL). (**C**) GST-pulldown assays analyzed by Western blotting to show molecular interaction of GST-CpoS C-ter with MBP-CpoS C-ter, confirming that the CC2 region was sufficient for tetramerization. (**D**) SEC-MALS profile of MBP-CpoS C-ter tetramer on Superose 6 Increase 10/300 Gl column. Normalized Kratky plot showing the flexibility of CpoS C-ter. PDDF fitting curves derived from the SAXS profile. SAXS profile for CpoS C-ter. The plot represents the logarithm of the scattering intensity (I, arbitrary units) as a function of momentum transfer (q, in Å^−1^).

Currently, the molecular architecture of CpoS is poorly understood, limiting our ability to understand the role it plays in modulating both other Chlamydial Incs and Rab GTPases. To address this, we conducted biochemical and biophysical analysis of the protein. CpoS, lacking the N-terminus and the bilobed hydrophobic domain (CpoS C-ter) (Fig. 4A), was expressed as an MBP-fusion in *E. coli*. Protein was isolated from bacterial lysates using affinity chromatography and was further purified using Superdex 200 at a flow rate of 0.5 mL/min. Coomassie staining and Western blotting of the elutions confirmed highly pure protein was obtained (Fig. S3A). Intriguingly, a higher molecular weight SDS-resistant species of CpoS C-ter was noted by Western blotting (Fig. S3A, asterisk), suggesting that it might oligomerize. Size-exclusion chromatography (SEC) of CpoS C-ter yielded three well-separated peaks, further suggesting CpoS forms higher-ordered structures (Fig. 4B). To rule out the possibility of protein aggregation, dynamic light scattering (DLS) and MP (Fig. 4B) were used to evaluate the nature of the proteins present within each peak. As shown in Fig. 4B, CpoS C-ter predominately formed tetramers (~242 kDa) and octomers (~488 kDa) in solution along with some MBP-tag and monomeric CpoS C-ter (~58 kDa). CpoS C-ter also formed distinct monomers, tetramers, and octomers in some of the fractions. Notably, CpoS oligomerization was pH dependent, with the tetramer being stable at neutral pH and dissociating into dimers and monomers in both acidic and basic buffer conditions (Fig. S3C).

**Fig 5:**
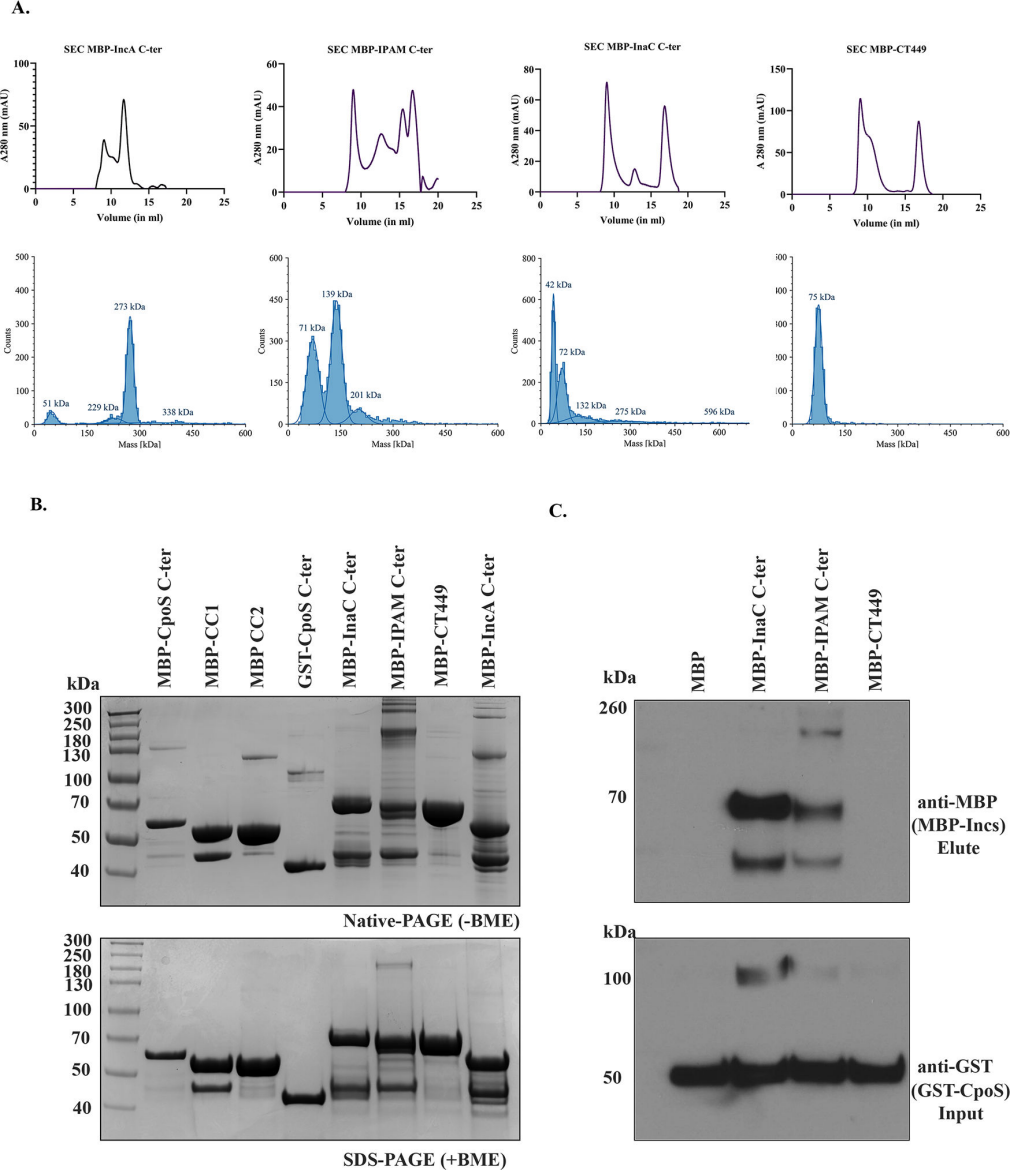
Intermolecular interactions between the C-terminal region of CpoS and other *C.t.* Incs. (**A**) Size exclusion chromatogram (SEC) and Mass photometry (MP) were used to evaluate the oligomerization properties of additional *C.t*. Inc proteins. The SEC profiles for IncA C-ter, IPAM C-ter, InaC C-ter showed multiple peaks, consistent with the presence of oligomeric species. In contrast, CT449 C-ter eluted primarily as a single peak and a void peak, suggesting a monomeric state. MP histograms confirmed oligomer formation for IncA C-ter (Fractions corresponding to 13 mL) IPAM C-ter (Fractions corresponding to 13 and 14 mL), InaC C-ter (Fraction corresponding to 17 mL), and monomers for CT449 (Fraction corresponding to 17 mL). (**B**) Native PAGE and SDS PAGE analyses of purified proteins further supported the presence of oligomers for the coiled-coil containing Incs. (**C**) In vitro-GST pulldown assays demonstrated that MBP-tagged InaC and IPAM with GST-tagged CpoS.

To determine whether assembly and oligomerization require CC1 or CC2 of CpoS, we expressed each region independently and analyzed purified protein samples by SEC and MP (Fig. S3A ;Fig. 4B). Similar to what was observed for CpoS C-ter, a higher molecular weight species was noted by Western blotting for CC2 but not CC1 (Fig. S3A, asterisk). The requirement of the CC2 domain for tetramer and octamer formation was further confirmed by SEC, MP, and native PAGE (Fig. 4B and 5B), revealing that only CpoS CC2 was capable of oligomerizing. We hypothesized that the formation of a CpoS tetramer required interactions between its CC2 region. To test this, we conducted *in vitro* pulldowns using GST-CpoS C-ter with MBP CpoS C-ter, MBP-CC1, MBP-CC2 regions. As predicted, CC2 was required for interaction with itself and tetramerization (Fig. 4C).

To further characterize the tetrameric structure of CpoS C-ter, SEC-MALS-SAXS was employed. The theoretical mass of the MBP-tagged Cpos C-ter tetramer is 234 kDa, as the molecular weight of the monomer, derived from the protein sequence, is 58 kDa. The absolute molecular mass of MBP-tagged CpoS C-ter, measured by MALS, was 254 and 263 kDa as measured by SAXS (Fig. 4D). These data are consistent with tetrameric assembly of CpoS C-ter. Following data reduction and buffer subtraction, the SAXS data were further processed using the BioXTAS RAW software (44). Analysis of the SAXS data indicated that the CpoS C-ter protein was monodisperse, as evidenced by the linearity of the Guinier plot (Fig. 4D). The normalized Kratky blot exhibited a maximum shift towards higher qRg values, and the *P*(r) function showed a peak at low radii followed by an extended tail, both suggesting an elongated shape for CpoS C-ter (Fig. 4D). The molecular weight of CpoS C-ter, estimated from SAXS data using various methods, was consistent with a tetrameric assembly: 262.8 kDa (by Vc method), 274.3 kDa (by Vp method), and 242.6 kDa (by Bayes method) (49–51). The Rg value derived from the Guinier approximation was 71.5 Å, while the *P*(r) analysis yielded an Rg of 76.7 Å and a maximum particle dimension (Dmax) of 303 Å. These parameters further support the elongated nature of the CpoS C-ter tetramer in solution. In summary, our SEC-MALS-SAXS analysis provides strong evidence for a stable, elongated tetrameric conformation of CpoS C-ter in solution, corroborating our previous biophysical characterizations (Fig. 4D).

### Other Inc proteins undergo oligomerization

To investigate whether other CC region-containing Inc proteins oligomerize, the purified proteins were analyzed by SEC, MP, and native PAGE. IncA, previously shown to form tetrameric structures, was included as a control (39). Our analysis revealed that IPAM C-ter and InaC C-ter formed dimers and trimers, while IncA C-ter formed a stable tetramer (252 kDa based on sequence), similar to the one seen for CpoS C-ter (234 kDa based on sequence). Consistent with previous reports using BATCH showing that InaC (CT813) can homo-oligomerize (23), our findings further support its ability to form oligomeric structures. As expected, CT449, which lacks a CC region, exclusively formed monomers (Fig. 5A). Oligomerization was further confirmed using native PAGE gel (Fig. 5B).

To confirm that CpoS binds directly to other CC region-containing Inc proteins, we performed *in vitro* pulldown assays, using GST-CpoS. Notably, MBP-InaC and MBP-IPAM, but not MBP-CT449 or MBP tag alone, bound to CpoS (Fig. 5C). These results are consistent with our IPs (Fig. 1B) and provide strong evidence that CpoS binds to multiple CC region-containing Inc proteins.

### CpoS-InaC interactions are required for InaC-mediated recruitment of Arf GTPases to the inclusion membrane

**Fig 6:**
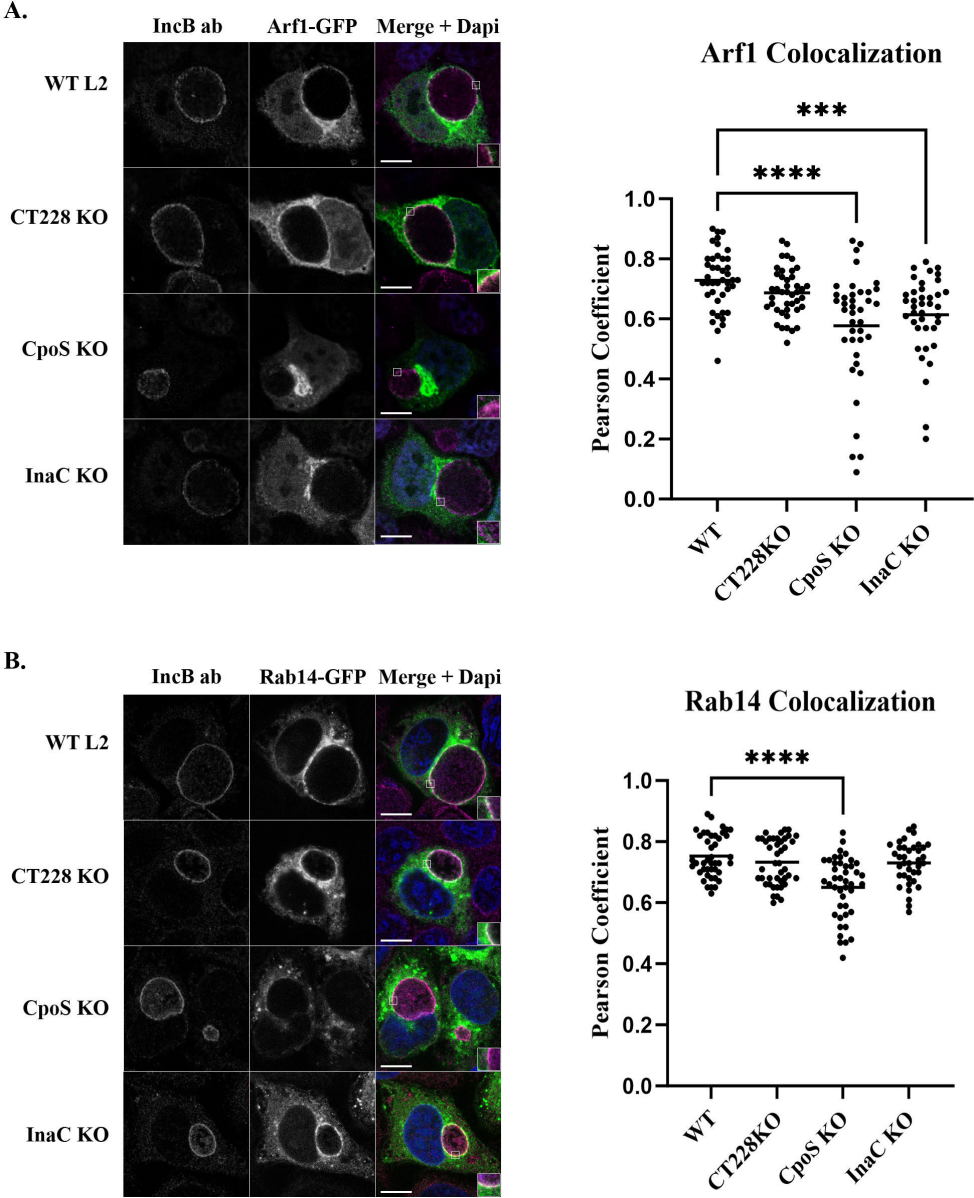
Loss of CpoS significantly affects Arf1 recruitment. (**A**) HeLa cells were transfected with pEGFP Arf1 or (**B**) pcDNA 3.1 Rab14 and infected with either WT L2, CT228*::bla*, *cpoS::bla*, or *inaC::bla C.t*. strains. Cells were MeOH fixed 24 hpi and processed for confocal imaging. Intact inclusions were stained using an IncB specific antibody and with anti-rabbit 594 secondary and DAPI. Scale bar denotes 20 µm. White boxes denote the area used for inset. Pearson correlation coefficients (R value) were calculated using ImageJ Coloc2 function. The graph is representative of 20 images per experiment. Data are representative of three independent experiments.

Given the results showing that multiple CC Incs bind to and colocalize with CpoS, we next sought to determine whether Inc-Inc interactions facilitate interactions with the cognate host factors. InaC is a well-characterized Inc that interacts with Arf GTPases 1 and 4 (19, 20), host proteins involved in ER-to-Golgi trafficking (52). To determine whether CpoS expression is required for InaC-mediated recruitment of Arf1 or Arf4, we assessed Arf1 localization to WT *C.t., cpoS::bla*, or *inaC::bla* inclusion membranes. As shown in Fig. 6A, Arf recruitment to the *cpoS::bla* mutant inclusion was impaired. To ensure this result was not an artifact caused by premature inclusion lysis, only intact inclusions, as indicated by IncB staining, were analyzed. Pulldown experiments further confirmed that CpoS does not bind directly to Arf1 (Fig. S4), suggesting that the impaired recruitment is due to an inability to engage InaC. Additionally, to rule out a general growth defect as the cause of the impaired recruitment, recruitment to the CT228*::bla* mutant was assessed, which showed no significant decrease in colocalization (Fig. 6A).

To determine whether Rab GTPase recruitment depends on CpoS binding to additional Inc proteins, we assessed Rab localization in cells infected with the *inaC::bla* or CT228*::bla* mutant strain. Rab recruitment to inclusions formed by the *inaC::bla* mutant showed no differences compared to wild type (Fig. 6B), indicating that while CpoS-InaC interactions are important for recruiting Arf GTPases, Rab GTPase recruitment occurs independently of Inc-Inc interactions. Similarly, infection with the CT228*::bla* mutant revealed no significant changes in Rab colocalization. These results demonstrate that CpoS-InaC interactions are specifically required for Arf1 recruitment, whereas Rab GTPase recruitment relies on a distinct mechanism. This underscores the cooperative nature of Inc proteins in directing host vesicular traffic to the inclusion.

## DISCUSSION

**Fig 7:**
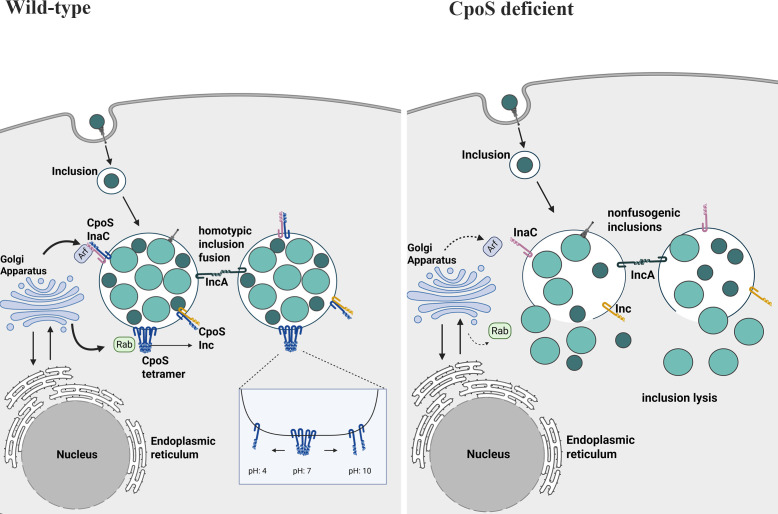
Model of Rab-CpoS-Inc role in coordinating host-pathogen interactions during *C.t.* infection. *In vitro*, CpoS oligomerizes, primarily as a tetramer, and interacts with multiple inclusion membrane proteins to regulate host membrane trafficking. Loss of CpoS disrupts the recruitment of Rab GTPases and indirectly reduces Arf1 localization to the inclusion. This impairment destabilizes the inclusion membrane, leading to premature lysis and the release of bacteria into the host cytosol.

In this study, we demonstrate that CpoS plays a multifaceted role during *C.t*. infection, interacting with both host Rab GTPases and various CC-region-containing Inc proteins (Fig. 7). Building on previous research, we show that CpoS specifically binds to itself and multiple other Inc proteins via its CC2 domain, a region distinct from the region required for Rab binding. Through these interactions, CpoS efficiently recruits diverse host vesicles to the inclusion, thereby supporting intracellular infection. Using biochemical and biophysical approaches, we show that CpoS forms higher-order structures, including tetramers and octomers, which we propose enables it to function as a scaffold for vesicle tethering and fusion, critical processes for delivering nutrient-rich cargo to the inclusion. The significance of this interactive network, centered on CpoS, is further highlighted by its essential role in maintaining the structural integrity of the inclusion membrane and evading the host immune response (21, 22, 25).

The chlamydial inclusion serves as a protected intracellular niche that allows *C.t*. to replicate while avoiding detection by the host’s innate immune response. *C.t*. is predicted to encode approximately 60 Incs, of which 37 have been confirmed to localize to the inclusion membrane during infection (10, 12, 53). Incs are strategically positioned on the cytoplasmic face of the inclusion, allowing them to interact with host factors while also engaging in self-interactions and interactions with one another. Genetic studies have shown that the absence of specific Incs, including CpoS, leads to destabilization of the inclusion membrane, premature lysis, exposure of bacteria to the cytosol, and failure to evade host defenses (22, 25, 54, 55).

While the function(s) of a limited number of Incs are defined (15, 17, 24, 55–57), CpoS is known to selectively recruit Rab GTPases to the inclusion membrane primarily through its CC1 region (14, 21, 22), although interactions with some Rab GTPases require additional C-terminal residues (14, 21). It is plausible that CpoS simultaneously interacts with both host Rab proteins and other Incs, as its interactions with Incs appear to be mediated by CC2. IncE, for example, an Inc protein that binds both host syntaxins and sorting nexins, has been shown to interact with multiple host factors in the same biochemical assay (58). This broad recruitment of Rab GTPases is crucial for capturing lipid-containing vesicles and for suppressing STING activation and interferon signaling (21). Notably, Rab35 is essential for CpoS-mediated modulation of the host immune responses, though its recruitment also depends on additional, yet unidentified, factors. Furthermore, CpoS-Rab interactions appear to prevent premature cell death during infection, suggesting that CpoS may regulate additional host pathways.

Early studies using bacterial two-hybrid assays and affinity purification mass-spectrometry (AP-MS) demonstrated that CpoS binds to several Inc proteins (23), and interactions with IPAM have been confirmed during infection (21, 22). Based on these observations, we hypothesized that CpoS may also bind to other CC-region-containing Incs, and here, we demonstrate interactions with CT226 and InaC. *C.t*. transcriptional activity can be broadly categorized into early, mid, and late stages, with effector proteins secreted in waves throughout the developmental cycle (59). Temporal expression provides a mechanistic basis for differential binding. CpoS expression peaks at 3 h post-infection but is maintained throughout the infection cycle; CT226 is detectable from ~3 hpi and persists throughout infection, whereas IPAM and InaC expression is not detected until mid-cycle stages of infection. This temporal expression suggests that CpoS may engage different partners as infection progresses, shaping the dynamics and functional consequences of Inc-Inc interactions. Importantly, previous studies have shown that different coiled-coil regions of CpoS have distinct functions in suppressing cell-autonomous defenses, with the CC2 region being especially important for inhibiting host cell death (21). This raises the intriguing possibility that Inc-Inc interactions are integral to establishing a stable replicative niche, potentially through coordinate acquisition of membrane material and the organization of the inclusion membrane.

Combining biochemical and biophysical methodologies to analyze the CC region containing Incs, we provide evidence that several Incs, including CpoS, can form higher-order oligomeric structures and engage in self-interactions. Their ability to oligomerize and interact with themselves and one another highlights the critical role Incs play in the formation of multi-protein complexes within the chlamydial inclusion membrane, which may enhance host protein recruitment to modify host cell physiology. Previous studies using two-hybrid assays suggested that Inc proteins, notably IncA, can oligomerize, with IncA requiring its SNARE-like domain for this process. Our biochemical data support this, revealing that CpoS and several other Incs form higher-order oligomers, dependent on their C-terminal region.

We propose a model in which Incs are inserted into the inclusion membrane via their transmembrane domains, while their coiled-coil regions form putative parallel four-helix bundles. This structural arrangement may enable membrane tethering and fusion, akin to eukaryotic SNARE-mediated vesicle docking and fusion. Notably, our FoldSeq analysis revealed that CpoS shares structural homology with t-SNARE proteins from *Trypanosoma cruzi* and v-SNARE proteins from *Arabidopsis thaliana*, despite minimal sequence similarity (Fig. S4). These findings suggest that CpoS may facilitate membrane interactions and fusion processes within the chlamydial inclusion in a manner similar to SNARE proteins. Given the central role of SNARE oligomerization in forming stable core complexes that drive membrane fusion, understanding how Inc proteins like CpoS exploit similar oligomerization strategies could provide valuable insights into the mechanisms by which *C.t*. manipulates host cellular processes for its benefit, highlighting a critical area for future exploration in host-pathogen interactions.

## SUPPLEMENTAL MATERIAL

Supplemental Figures iai.00548-25-s0001.docx Figures S1 to S5.

Table S1 iai.00548-25-s0002.docx Primers.

ASM does not own the copyrights to Supplemental Material that may be linked to, or accessed through, an article. The authors have granted ASM a non-exclusive, world-wide license to publish the Supplemental Material files. Please contact the corresponding author directly for reuse.
