# Supplementary material for: CpoS-Inc interactions facilitate host cell modulation during Chlamydia trachomatis infection
Source: Infect Immun. 2025 Nov 18;93(12):e00548-25. doi: 10.1128/iai.00548-25 (PMC12707108; doi:10.1128/iai.00548-25)
Supplement: Supplemental Figures — Figures S1 to S5. [file iai.00548-25-s0001.docx]

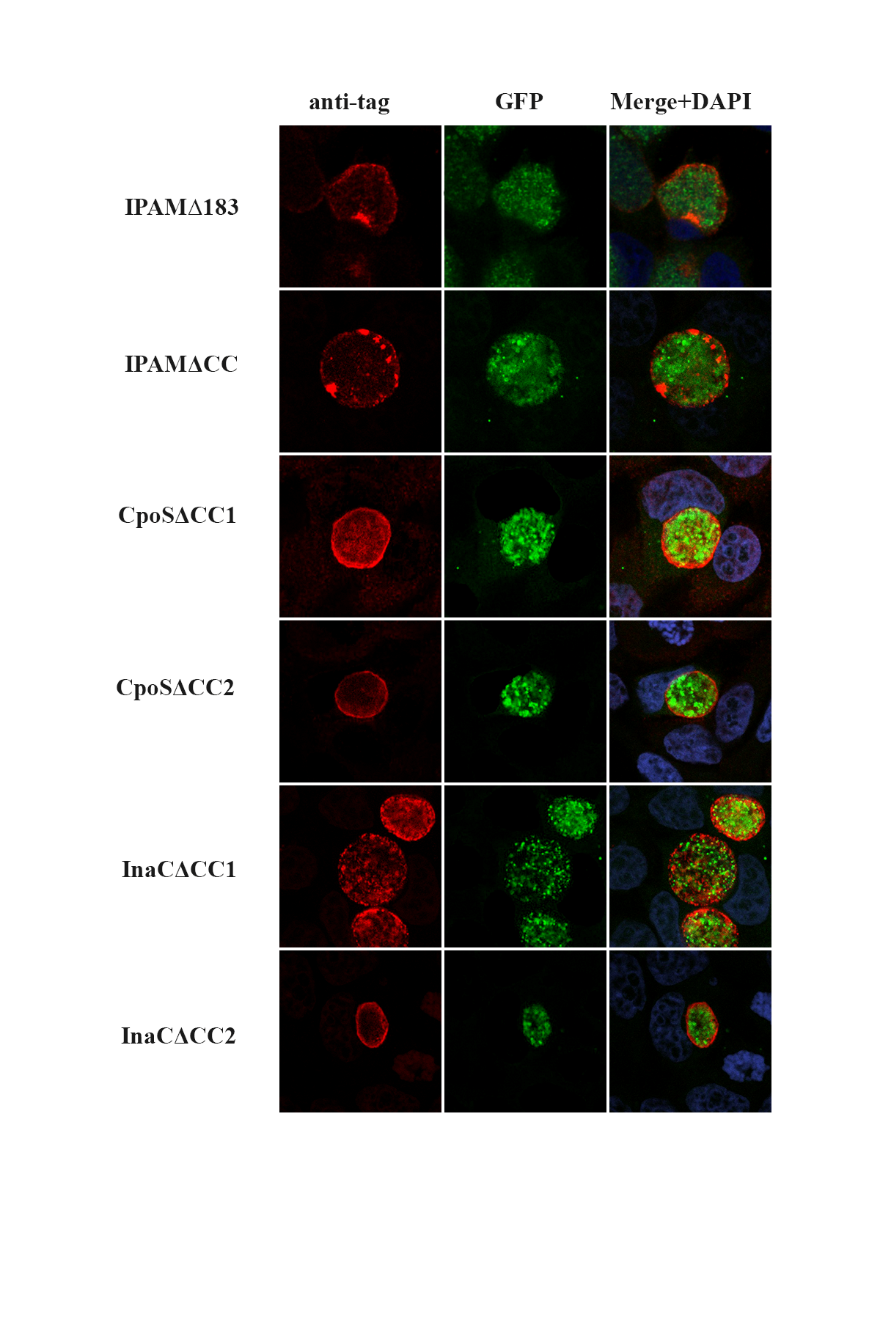


Supplementary Figure 1: Truncated Inc constructs retain inclusion localization. HeLa cells were infected with *C.t.* strains expressing FLAG-tagged Inc constructs. Expression was induced at time of infection. At 24hpi, cells were formaldehyde fixed and stained using an anti-FLAG (red) and DAPI (blue). The pBomb4 vector encodes GFP, enabling visualization of transformed *C.t.* during infection.

**
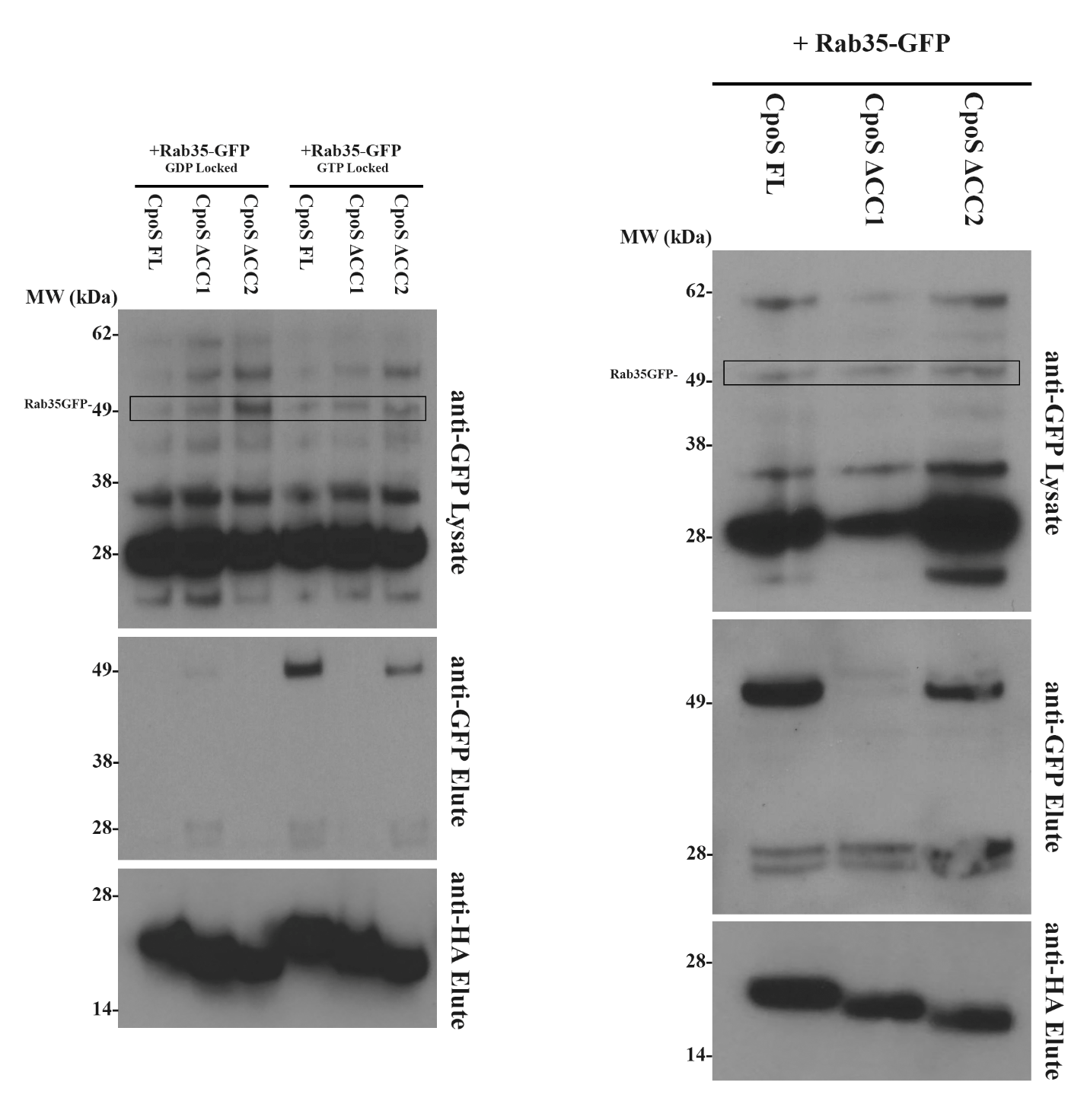
**

**Supplementary Figure 2**: CC1 of CpoS is necessary for Rab binding**.** HeLa cells were transfected with pcDNA3.1 Rab35 and infected with *C.t.* expressing either pBomb4-tet-CpoS FL, *C.t.* pBomb4-tet-CpoS ΔCC1, or *C.t.* pbomb4-tet-CpoS ΔCC2. Expression of CpoS was induced at the time of infection using aTc. At 24hpi, cells were lysed and CpoS constructs were immunoprecipitated using anti-HA magnetic beads. Samples were analyzed by western blotting to detect Rab35 binding (anti-GFP) and to confirm the IP (anti-HA). Data are representative of 2 experiments.

**
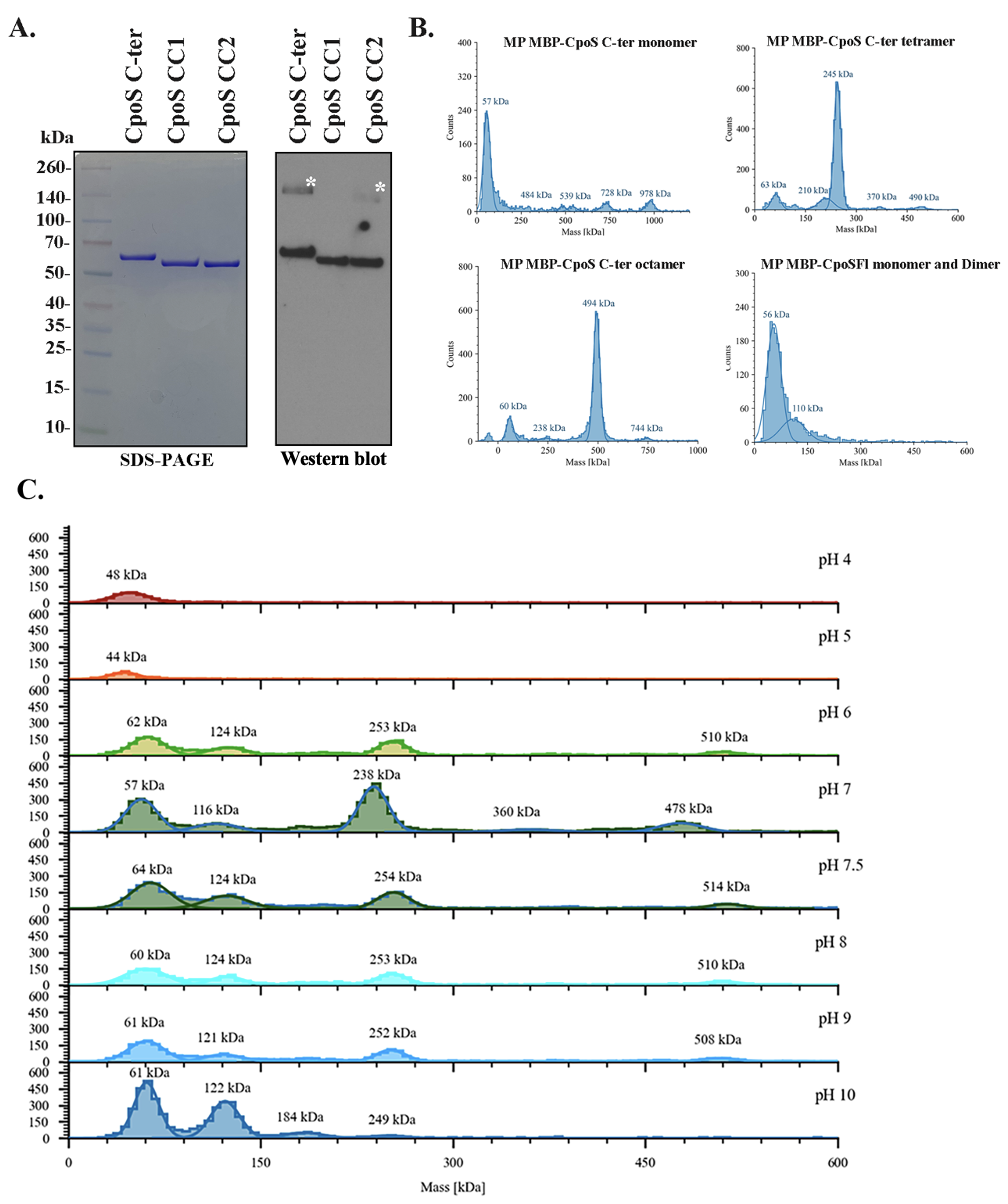
**

**Supplementary Figure 3:** Oligomerization and pH-Dependent stability of CpoS C-terminal and Coiled-Coil domains (A) Reducing SDS-PAGE analysis of MBP- tagged pure proteins of CpoS C-ter, CC1, and CC2 domains confirms SDS-resistant higher oligomers for CpoS C-ter and CC2 domains, but not for the CC1 domain. Coomassie (left) and western blot (right). (B) MP histogram showing the oligomerization properties of MBP-tagged CpoS C-ter protein, revealing distinct species of monomers, tetramers and octamers in solution following SEC. (C) pH dependent dissociation of CpoS C-ter tetramers into distinct monomers under acidic and basic conditions, as shown by MP histograms. Vertical stacked MP histograms of the tetramerized form of CpoS C-ter illustrate dissociation in acetate buffers (pH 4, 5, and 6) and Tris buffers (pH 7, 7.5, 8, 9, and 10) as pH deviates from neutral. The tetramers are most stable at pH 7 and tend to dissociate under more acidic or basic conditions.


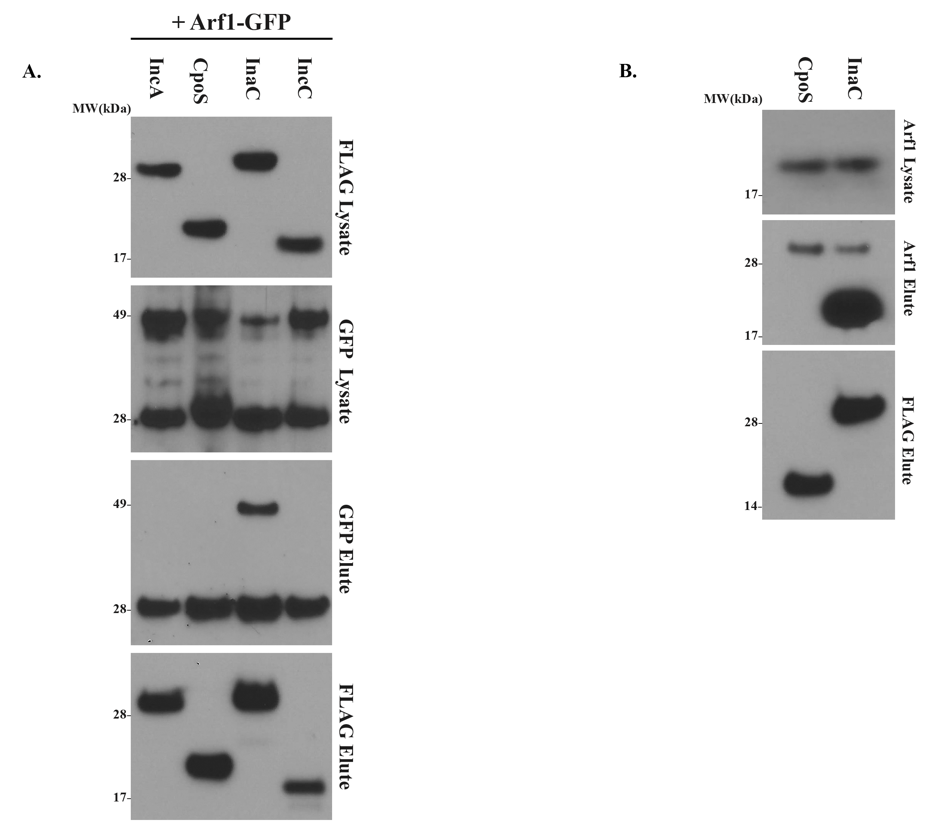


**Supplementary Figure 4:** CpoS does not bind Arf1. (A) HeLa cells were transfected with pEGFP Arf1 and infected with either *C.t.* pBomb4-tet-InaC-FLAG, *C.t.* pBomb4-tet-CpoS-FLAG, *C.t.* pBomb4-tet-IncA-FLAG, or *C.t.* pBomb4-tet-IncC-FLAG. (B) HeLa cells were infected with *C.t.* pbomb4-tet-CpoS-FLAG or *C.t.* pbomb4-tet-InaC-FLAG. (A, B) Expression was induced at time of infection. At 24hpi, cells were lysed and Incs were immunoprecipitated from cell lysates using FLAG-magnetic beads and samples were analyzed by western blotting to detect Arf1 binding using anti-GFP (A) or anti-Arf 1 antibodies (B). Data are representative of 2 experiments.


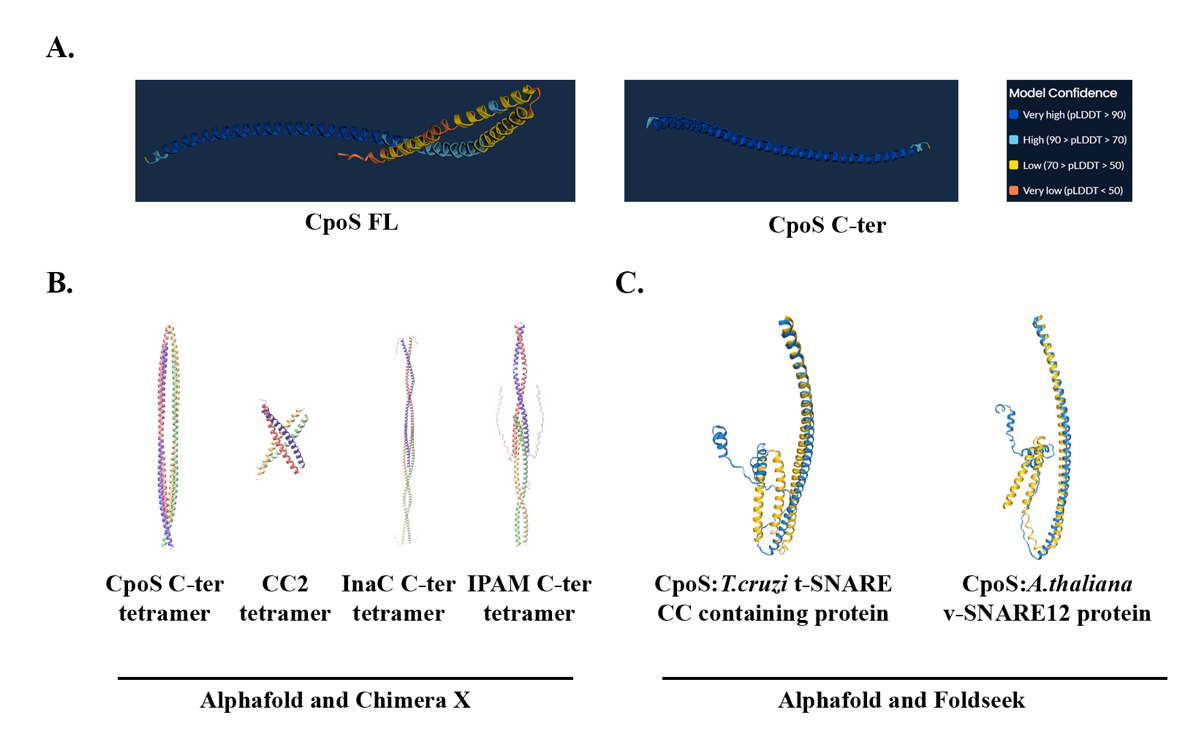


**Supplementary Figure 5:** (A) Alphafold predictions of CpoS full length and CpoS C-ter (devoid of two transmembrane domains) show long alpha helices comprising both coiled-coil domains. (B) Alphafold predictions of tetramer forms of CpoS C-ter, CC2 domain, InaC and IPAM (C) Foldseek data showing CpoS superimposed against t-SNARE coiled-coil homology domain containing protein of of *T.Cruzi* and v-SNARE12 protein of *A.thaliana*.
