# Supplementary material for: CpoS-Inc interactions facilitate host cell modulation during Chlamydia trachomatis infection
Source: Infect Immun. 2025 Nov 18;93(12):e00548-25. doi: 10.1128/iai.00548-25 (PMC12707108; doi:10.1128/iai.00548-25)
Supplement: Table S1 — Primers. [file iai.00548-25-s0002.docx]

Table S1: Primers used for completion of this study. Nucleotides in bold correspond to the restriction site. Nucleotides in blue correspond to the FLAG-tag, purple correspond to the HA-tag.

| **Primer Name** | **Sequence** |
| --- | --- |
| ***C.t.* Expression** | |
| CT229 NotI F | CC**GCGGCCGC**ATGAGCTGTTCTAATGTTAATTCAGGT |
| CT229 HA KpnI R | CC**GGTACCctaagcgtaatctggaacatcgtatgggta**TTTTTTACGACGGGATGCC |
| CT229 HA SLD2 removed SalI R | CC**GTCGACctaagcgtaatctggaacatcgtatgggta**ATGACGAATTTCTTGTTTAGCA |
| CT813 NotI F | CC**GCGGCCGC**ATGACTACTCTTCCCAATACTT |
| CT813 FLAG KpnI R | CC**GGTACCttacttatcgtcgtcatccttgtaatc**TATCGAACCACGTCTTCCTG |
| CT813 del CC1 342bp FLAG KpnI R | CC**GGTACCttacttatcgtcgtcatccttgtaatc**GGATTTTTGCTCTATCCAAGACT |
| CT813 del CC2 414bp FLAG KpnI R | CC**GGTACCttacttatcgtcgtcatccttgtaatc**TGAAAGGAATTCCACTTCTGAT |
| CT223 NotI F | CC**GCGGCCGC**ATGGTGAGTTTAGCATTAGGGAC |
| CT223 FLAG KpnI R | CC**GTCGACttacttatcgtcgtcatccttgtaatc**CACCCGAGAGCCGTAATT |
| CT223 del CC 294bp FLAG KpnI R | CC**GGTACCttacttatcgtcgtcatccttgtaatc**AGATCTCAAGCAACAACTACAT |
| CT223 549bp FLAG KpnI R | CC**GGTACCttacttatcgtcgtcatccttgtaatc**CTGTTTCTCTTTAGCAGGCAGGA |
| CT226 NotI F | CC**GCGGCCGC**TTGCGAAATAGAGGCGC |
| CT226 FLAG KpnI R | CC**GGTACCttacttatcgtcgtcatccttgtaatc**TCTCAGACTTTCTTCCAATA |
| CT226 del CC 363bp FLAG KpnI R | CC**GGTACCttacttatcgtcgtcatccttgtaatc**TAACACTAATCCAGGAGTCACGG |
| CT222 NotI F | CC**GCGGCCGC**ATGCGTTGCTGTTGTGTTC |
| CT222 FLAG KpnI R | CC**GGTACCttacttatcgtcgtcatccttgtaatc**GTGGAATACACTAATTGCTT |
| CT222 del CC 297bp FLAG KpnI R | CC**GGTACCttacttatcgtcgtcatccttgtaatc**AGACACTCGCTTCATACGCA |
| IncD NotI F | CC**GCGGCCGC**GTGAGAAATGAGATCTGGCTAAA |
| incD FLAG SalI R | CC**GTCGACttacttatcgtcgtcatccttgtaatc**GCTCGCCCCTTTTTTACTCACC |
| IncB NotI F | CC**GCGGCCGC**ATGGTTCATTCTGTATACAATTC |
| IncB KpnI FLAG R | CC**GTCGACttacttatcgtcgtcatccttgtaatc**TTCTTGAGGTTTTGTTGGG |
| CT288 NotI F | CC**GCGGCCGC**ATGGTTTATTTTAGAGCTCATCA |
| CT288 KpnI FLAG R | CC**GTCGACttacttatcgtcgtcatccttgtaatc**GTGATTATCTAACAGGTATT |
| CT224 NotI F | CC**GCGGCCGC**ATGAGTTTTGTTGGAGATAGTGT |
| CT224 KpnI FLAG R | CC**GTCGACttacttatcgtcgtcatccttgtaatc**ATCATTGGGAAAAATTGAGT |
| CT228 NotI F | CC**GCGGCCGC**ATGAGTACTACTATTAGCGGAGA |
| CT228 KpnI FLAG R | CC**GTCGACttacttatcgtcgtcatccttgtaatc**AGAAGCTTGGTTAGCGTCTA |
| CT449 NotI F | CC**GCGGCCGC**ATGAAATTACCAGAAGTGAGTT |
| CT449 KpnI FLAG R | CC**GTCGACttacttatcgtcgtcatccttgtaatc**CTGAATAGGCGCTTCAGAAG |
| IncA NotI F | CC**GCGGCCGC**ATGGACAAAATTAAGAAAATAGC |
| IncA KpnI FLAG R | CC**GTCGACttacttatcgtcgtcatccttgtaatc**GGAGCTTTTTGTAGAGGGTG |
| **Protein expression** | |
| Truncated CT229 NotI FP | GACGC**GCGGCCGC**CTTGCTAGAGCACGAAGTCGT |
| Truncated CT229 SalI RP | GACGC**GTCGAC**CTATTTTTTACGACGGGATGCCTGC |
| CT229 CC1 NotI FP | GACGC**GCGGCCGC**TTAGGCCTTGCTAGAGCACG |
| CT229 CC1 SalI RP | GACGC**GTCGAC**CTTACTTACAGCTCGACGAGC |
| CT229 CC2 NotI FP | GACGC**GCGGCCGC**CAGACAGAAAGGTTTAAACAG |
| CT229 CC2 SalI RP | GACGC**GTCGAC**TTTTTTACGACGGGATGCCTG |
| FLCT813 NotI FP | GACGC**GCGGCCGC**ATGACTACTCTTCCCAATACT |
| FLCT813 SalI RP | GACGC**GTCGAC**CTATATCGAACCACGTCTTCCTGG |
| FLCT223 NotI FP | CC**GCGGCCGC**ATGGTGAGTTTAGCATT |
| FLCT223 SalI RP | CC**GTCGAC**CTACACCCGAGAGCCGTAATTG |
| FLIncA Not1 FP | CC**GCGGCCGC**ATGACAACGCCTACTCTAATCG |
| FLIncA SalI RP | CC**GTCGAC**CTAGGAGCTTTTTGTAGAGGGTG |
| FLIncB NotI FP | CC**GCGGCCGC**ATGGTTCATTCTGTATACAATTC |
| FLIncB SalI FP | CC**GTCGAC**CTATTCTTGAGGTTTTGTTGGG |
| CT223 truncated NotI F | CC**GCGGCCGC**ATGAATACATGTAGTTGTTGCTT |
| CT223 truncated SalI R | CC**GTCGAC**CTACACCCGAGAGCCGTAATTG |
| CT449 NotI F | CC**GCGGCCGC**ATGAAATTACCAGAAGTGAGTT |
| CT449 SalI R | CC**GTCGAC**TTACTGAATAGGCGCTTCAGAAG |
| CT813 truncated Not F | CC**GCGGCCGC**ATGCAAGTTGAGAAATCTC |
| CT813 truncated SalI R | CC**GTCGAC**CTATATCGAACCACGTCTTCCTG |
| IncA truncated NotI F | CC**GCGGCCGC**ATGCAGAAAACCGCTAATC |
| IncA truncated SalI R | CC**GTCGAC**CTAGGAGCTTTTTGTAGAGGGTG |
